# Healthcare professionals’ attitudes towards digital health interventions and perspectives on digital health inequalities in cardiometabolic care: a qualitative study

**DOI:** 10.1136/bmjopen-2024-091018

**Published:** 2025-02-26

**Authors:** Mel Ramasawmy, David Sunkersing, Lydia Poole, Ann Blandford, Paramjit Gill, Kamlesh Khunti, Shivali Modha, Kiran Patel, Henry Potts, Madiha Sajid, Nushrat Khan, Amitava Banerjee

**Affiliations:** 1 Institute of Health Informatics, University College London, London, London, UK; 2 Wolfson Institute of Population Health, Queen Mary University of London, London, UK; 3 University of Surrey, Guildford, UK; 4 UCLIC, University College London, London, UK; 5 University of Warwick Medical School, Coventry, UK; 6 Diabetes Research Centre, University of Leicester, Leicester, UK; 7 Patient and Public Involvement Representative, DISC Study, London, UK; 8 Centre for Health Informatics and Multiprofessional Education, UCL, London, UK; 9 School of Public Health, Imperial College London, London, UK

**Keywords:** Telemedicine, Cardiovascular Disease, Health Equity, DIABETES & ENDOCRINOLOGY

## Abstract

**Abstract:**

**Background:**

The ue of digital health interventions (DHIs) for the management of cardiometabolic diseases has increased but may exacerbate existing health inequalities. Healthcare professionals (HCPs) play a vital role in recommending and supporting healthcare users to use these tools. There is a need to understand the role of HCPs in managing the implementation of digital health in communities at risk of health inequalities.

**Objective:**

To explore the views of HCPs regarding digital health and its impact on health inequalities, focusing on cardiometabolic diseases and the South Asian population in the UK.

**Design:**

We conducted online semi-structured interviews and focus groups with HCPs. These explored HCPs’ experiences and attitudes towards digital health, their perceptions of healthcare users’ barriers and facilitators to use such tools, as well as digital inequalities among specific healthcare user groups, and the impact of the COVID-19 pandemic on their practice in relation to digital health. After informed consent, transcription and coding, a reflexive thematic approach was taken for analysis.

**Setting:**

Primary, community and secondary care provided for cardiometabolic disease in the UK.

**Participants:**

HCPs in general practice (n=3), cardiometabolic specialities (n=3), pharmacy (n=4) and other practices (n=8).

**Results:**

HCPs recognised the potential benefits of DHIs to improve access and delivery of care and healthcare user outcomes but described several barriers to successful implementation. HCPs demonstrated a good understanding of the challenges their healthcare users face in relation to wider inequalities, barriers to health behaviours and healthcare access, and digital health. Of particular concern was the impact of increasing reliance on digital interventions in healthcare on the exclusion of some population groups. Participants recommended improvement of the design and implementation of DHIs offered to healthcare users through working with at-risk populations throughout the process. Finally, participants emphasised the importance of ensuring non-digital services remained available to ensure equitable access to health and social care.

**Conclusions:**

HCPs described the complexities of delivering care to underserved communities. DHIs were identified as a potential way to improve health outcomes for some, while over-reliance risked exacerbating inequalities. HCPs made recommendations related to design, implementation and engaging target populations and provided practical examples to address digital health inequalities, such as working with other sectors to take a community approach.

**STRENGTHS AND LIMITATIONS OF THIS STUDY**

- The use of qualitative focus groups and interviews elicited rich data about HCP experiences and perceptions of digital health inequalities.
- A range of HCPs were included in the study, which allowed us to explore the use of DHIs along the cardiometabolic diseases care pathway.
- Many of the HCPs recruited were from a minority ethnic background. Although not deliberate, this might reflect interest or confidence in speaking about health inequalities and ethnicity—a limitation of the study.

## Background

 Digital health interventions (DHIs), such as mobile phone applications (apps), wearables and websites, have the potential to improve healthcare user knowledge and outcomes and save healthcare costs.^1^,^4^ The UK government is investing significantly in digital transformation and innovation,^5^ and healthcare users can now use their online National Health Service (NHS) account to access NHS and commissioned services such as managing bookings and referrals, accessing healthcare records and using online pharmacies.^6^

Healthcare professionals (HCPs) play a pivotal role in the introduction and uptake of DHIs. Previous studies have shown variation in the digital health competence of HCPs^7^ and highlighted barriers and facilitators to HCP use of digital health. This includes infrastructure, technical barriers, training, evidence about technology effectiveness, concerns about workload and individual level barriers, such as resistance to change or concerns about losing human interaction.^8^ There has also been some exploration of HCPs’ attitudes and behaviours in relation to apps for specific conditions such as depression^9^ or healthcare users use of wearables.^10^

The implementation of DHIs may exacerbate existing health inequalities, for example, by age, ethnicity, socio-economic status and health conditions.^11^ There is therefore a need to understand the role of HCPs in managing the implementation of digital health in communities at risk of health inequalities. The NHS has invested in a range of DHIs around prevention and management across primary, secondary and community settings, including supporting lifestyle change, remote monitoring and rehabilitation.^12^,^14^ However, it is important to further explore whether and how such digital interventions support HCPs in the care pathway in different healthcare settings and how digital tools could be developed and promoted by taking potential inequalities into account. Given the South Asian population in the UK experiences poorer cardiometabolic health outcomes and is more likely to experience barriers to digital inclusion,^15 16^ we aimed to understand HCPs’ perceptions of digital health and health inequalities, focusing this healthcare user population as a case study.

## Methods

### Study design

A qualitative approach encompassing interviews and focus groups was taken. The study received ethical approval from NHS London—Brent Research Ethics Committee (IRAS 261047). Study reporting was completed in line with COREQ guidelines (see online supplemental material).

### Focus groups and interviews

Recruitment of HCPs within the UK took place via circulation of information about the study in primary care practices, professional networks and organisations and using snowball sampling methods.^17^ The interviewer(s) were not known to the participants prior to the discussion. Inclusion criteria were HCPs employed in a health or care role in primary, secondary or community care settings, with experience of managing individuals with cardiovascular disease and/or diabetes. Experience of using DHIs was desirable but not essential, to support understanding of barriers and facilitators across healthcare services. If participants were not available to attend an online focus group (lasting 1 hour), interviews were offered instead to enable participation (lasting between 30 min to 1 hour). Focus groups and interviews took place between April and December 2022, and new data collection was stopped when we had spoken to a range of HCPs across primary, secondary and community care, and no new themes emerged from the discussions.

Before each focus group or interview, participants provided written informed consent, with any queries addressed by the research team. The semi-structured discussions explored professionals’ experiences and attitudes towards digital health, perceptions of healthcare users, barriers and facilitators to use, whether they perceived any populations to be particularly at risk of digital inequalities and the impact of the COVID-19 pandemic on their practice in relation to digital health (full details are provided in online supplemental material 1). Participant contributions were summarised to confirm understanding during the focus groups and interviews. Participants were offered a £50 retail voucher for their time.

Focus groups and interviews were conducted by MR and NK, who are both experienced qualitative researchers. Discussions were recorded and transcribed verbatim using Microsoft Teams and were checked and anonymised by the research team afterwards. A reflexive thematic approach was taken for analysis.^18^ After familiarisation, transcripts were coded by MR and NK using Microsoft Excel. An iterative process of coding, testing and revision of codes was completed by MR, NK and DS, and codes were organised into themes through discussion between MR, NK and DS. The team involved in interviews and analysis (MR, NK and DS) are early career researchers who are familiar with South Asian culture, and the wider co-author team includes people with lived experience, clinicians and researchers with expertise in cardiometabolic disease and supporting underserved populations.

### Patient and public involvement

PPI contributors with lived experience, or experience as carers, and from a South Asian background were involved in the study design, creation of participant-facing materials, interpretation of findings, drafting of outputs and co-produced dissemination materials.

## Findings

### Participant characteristics

18 HCPs working across primary, community and secondary care in the UK were recruited (see table 1). In total, there were three focus groups (n=3–6) and four individual interviews. For participants who provided demographic data (n=16, 89%), the mean age was 38 years, and 10 were females (63% of those who provided details). The majority of participants were from a South Asian ethnic background (n=13, 72%), which was not planned, but may reflect interest in the subject and confidence in speaking about ethnic inequalities in health. Digital literacy of participants was not documented.

**Table 1:** Participant demographics

	N (%)
Age	
20–29	3 (17%)
30–39	8 (44%)
40–49	3 (17%)
50–59	1 (6%)
60–69	1 (6%)
Not provided	2 (11%)
Gender	
Female	10 (56%)
Male	5 (28%)
Other/not provided	3 (17%)
Ethnicity	
Asian/Asian British	13 (72%)
White	3 (17%)
Other/not provided	2 (11%)
Religious beliefs	
Christian	1 (6%)
Hindu	6 (33%)
Muslim	4 (22%)
Sikh	2 (11%)
None	3 (17%)
Other/not provided	2 (11%)
Role	
General practice	3 (17%)
Pharmacy	4 (22%)
Specialist doctor or nurse—diabetes	2 (11%)
Specialist doctor or nurse—ardiology	1 (6%)
Doctor or nurse—other	3 (17%)
Dietician or nutritionist	2 (11%)
Other health role	3 (17%)

### HCPs’ attitudes and experiences of DHIs

Participants described various digital health approaches in use in primary and secondary care around monitoring, information provision and appointment or medication administration, with a range of complexities. Specific examples related to CMD monitoring ranged from providing healthcare users with low-cost blood pressure devices to the NHS ‘Heart failure @ home’ programme.^19^ There was a perception of different levels of acceptability of the implementation of digital in health within and between primary and secondary care, different specialities and within pharmacy where healthcare users often sought additional advice.

HCPs highlighted potential benefits of digital approaches in the NHS, such as collecting data that supported consultations, speeding up diagnosis and treatment and managing waiting lists. They praised the positive impacts of technologies on healthcare user self-management and outcomes, such as continuous glucose monitoring, and the potential for improved communication and follow-up of relevant information, through SMS messages, and links to leaflets, videos and websites.

…when patients are having appointments with clinicians whereby we only have 10 to 15 min to discuss an issue, then we tend to use these particular types of leaflets and videos (sent by text message) as a bit of a supplementation to what we've discussed in the consultation. (P5, Pharmacist)

However, they also highlighted that those benefits were not yet always realised, for example, on a platform designed for healthcare users to undertake and record their own blood pressure readings, one clinician noted that “about 60–70% of the time you’ve got to chase them anyway… (it) wasn't as beneficial as we'd have liked.” (P10, Consultant in Diabetes). Another reflected on the potential risk of harm, such as individuals being given access to their electronic health records without adequate support:

… not only do people look at the record and, you know, start to query what the doctor’s written, or they don't really understand what the doctor’s written… (P18, Junior doctor)

#### HCPs’ perspectives on DHI acceptability to healthcare users

Participants described mixed attitudes of healthcare users towards DHIs, some of which were dependent on the type and immediacy of the health condition. For example, healthcare users were described to be happy to have routine appointments over the phone but would prefer to see an HCP face-to-face for new conditions or where required, such as diabetic foot checks. Participants reported that healthcare users with diabetes were very engaged by continuous glucose monitoring, seeing it as preferable to finger pricking. HCPs reported that other interventions such as DAFNE (‘Dose Adjustment For Normal Eating’, an NHS Type 1 diabetes education programme)^20^ and digital weight loss programmes had a more mixed uptake and saw high drop-off after referral.

They noted that healthcare users were often already using smartphones for things of interest to them, such as speaking to family members, and that the increased use of digital applications in non-health contexts had also increased acceptability in health contexts. Several HCPs spoke about how, in contrast to their expectations, healthcare users felt enabled by technology and wanted to own and use their health data and access the latest technology to help manage their condition.

“(With continuous glucose monitoring) - the view was patients weren't going to be very interested in this - but you know that I think all of our experience it’s the patients are the ones driving it and (the) NHS - we have to catch up! (P2, Academic Clinician (Nursing))

However, they also noted healthcare user concerns about data privacy and that this overlapped with other hesitancy to engage with healthcare, such as vaccine hesitancy.

…I guess you can quite easily correlate (vaccine hesitancy in minority ethnic populations) with some of the interventions that require people to put in personal information. There might be this element of, ‘well, what on earth are they gonna do with this information? (P5, Pharmacist)

There was also a view that healthcare users perceived remote care as of lower quality or represented HCPs avoiding seeing people or ‘*fobbing them off*’ to save time or money. Clinicians noted that an important factor in overcoming this was the healthcare user relationship, reinforcing the use of digital tools and supporting effective use through follow-up.

I would also want to make sure that it could be followed up effectively… It’s not just something that you give to them and say go away and do this for 6 weeks. It’s something that you can check in and see that they're actually following it as… it was intended. (P18, Junior doctor)

#### Lack of evidence to support recommending DHIs to individuals with cardiometabolic disease

When recommending DHIs to healthcare users, professionals drew on their experience and knowledge, mentioning that there was variability between clinicians. Examples of the types of tools clinicians felt comfortable with recommending included simple commercial apps to improve diet and physical activity levels. Participants spoke about the importance of shared decision-making in promoting adherence and gave examples of how a decision to use DHIs was often driven by healthcare users’ interest in DHIs and their exposure through friends, family and media.

It’s quite difficult to keep up with (the pace of change in digital health) - you know, often people come to us and say, ‘Well, can I have this device?’ that I've never heard of… [P4, Consultant in diabetes]

I would volunteer (DHIs) for patients that were struggling or the patients that are saying… ‘Can you advise me on something?’ - and I can say well, I'm familiar with these (commercial diet and exercise apps). And it is anecdotal feedback from patients, a lot of patients tell me… This app’s good. (P6, GP)

All participants spoke about how COVID-19 restrictions and the need to deliver care remotely had an impact on digital health offerings, uptake by their healthcare user populations and openness of the healthcare system to digital tools. Without sufficient NHS services in place for some remote monitoring tools, clinicians made recommendations to use commercial apps to support diagnosis (eg, for atrial fibrillation). One GP explained how uptake of a commercial platform ‘dramatically increased’ during the pandemic and how this rapidly changed how they communicated with healthcare users:

[Pre-pandemic] we never sent texts, we never asked for text back from patients, photos and information, we never did video consultations… What we would probably not have done in 5–6 years, we did in a couple of weeks. [P6, GP]

… it’s not very easy to diagnose atrial fibrillation over the phone. So we just have to rely on patient symptoms. But there’s [now] such a long wait to have… Holter monitoring… So I've been recommending to patients because they are obviously feeling quite unwell to buy (commercial DHI). And that really has made a difference (to diagnosis and initiating treatment). (P2, Academic Clinician (Nursing))

However, participants felt that a lack of evidence about the efficacy of DHIs impacted the advice they were able to give to healthcare users, for example, not being able to make a recommendation directly, with one consultant describing themselves as ‘*hamstrung*’ by NICE recommendations (P4). They wanted more joined-up commissioning, such as NHS-driven DHI infrastructure, which would enable them to select appropriate tools from a trusted source, know that this was funded in their region and which would ensure data was shareable between parts of the NHS. Integrated systems which could be accessed via *‘one entry point’* were seen as beneficial *‘to simplify it for the health professional, but also for the patient as well’* (P10). Overall, HCPs currently felt restricted and needed more support, training and information to make recommendations to healthcare users safely.

### Perceptions of inequalities and intersection with digital exclusion

HCPs had a nuanced understanding of the challenges and needs of their healthcare user populations, the intersecting factors that contributed to health inequalities, and how this impacted uptake and engagement with digital health. This included reflections on the impact of the cost-of-living crisis, the difficulties of providing care around prevention and management of cardiometabolic diseases in deprived communities and the lack of resources, such as interpretation, to support healthcare users facing inequalities in access.

(Rare diabetic emergencies) seem to be becoming more prevalent and it’s as a result of COVID and sort of the pressure on people. I think also going back to heating vs eating that is a huge problem, you know, so patients can’t afford bus fares or train fares to come to hospital, and that’s gonna be a big issue, which no one’s really sort of considered. So we roll out 5G. Yeah, great. But no one can - no ones going to be using the technology because they're afraid of more costs. (P2, Academic Clinician (Nursing))

HCPs also discussed the impact of the changing food environment on population health as something beyond their scope. This requires intervention at a policy and local government level.^21^

Where the (primary care practice is based)… 20 years ago had a mixture of shops, and now it’s just 90% takeouts… So you know, so one of the biggest battles would be fast food. More than anything else and you don't know what one can do about that. (P15, GP)

Participants described inequalities they observed in their areas of work as being more related to social deprivation than specific ethnic or cultural groups. An example of this was that new migrants to the UK (such as those from Eastern Europe who arrived after Brexit) were showing the same patterns of health problems and lack of engagement with healthcare services as previous generations of South Asian migrants.

HCPs also reflected on their positionality (their social identities) and how this affected their ability to engage with diverse healthcare users. For example, one South Asian GP, speaking about family dynamics and their role in promoting health behaviour, explained how he can engage the whole family in health changes, using his familiarity with South Asian cultural norms: *‘… the children generally are quite involved… and they usually live together so… I also tell them that if their mum or dad is diabetic, then they're also more likely to have diabetes if it’s the son or the daughter. So then that way it kind of helps to try and improve everyone’s diet altogether so that they're quite keen on that’* (P6). He reflected on a recent appointment with another individual, who came in on his own and *‘said he didn't really want to bother his children to make, like, special food just for him’* and how, in other cultures and family dynamics, he was not able to use that same strategy.

Several participants commented on the intersection between deprivation and health literacy and how these might interact with potential benefits of digital implementation. For example, one primary care pharmacist explained:

The people that… don't have so much money. They definitely struggle with, first of all understanding like the diabetes. And then I have to spend a lot more time with them to explain why we need to get (the condition) under control. And I think that’s why they're less likely to be proactive and you know, want to have these apps and do these things. (P7, Primary Care Pharmacist)

Some participants described typical factors related to digital exclusion such as age, generation, language spoken, literacy and education, cost and access to devices, as well as other specific groups at risk such as those leaving prison, those with physical barriers (such as arthritis and sight or hearing impairment) and those with learning difficulties. However, others highlighted that widely held perceptions of inequalities did not necessarily match what was observed in practice.

I've been quite surprised at, you know, older Bangladeshi diabetic women who come and see me online via Attend Anywhere, often with maybe one of their relatives helping them out and so on. Where I've always felt, actually these are the sort of people that might not want to engage online, but actually I've been very pleasantly surprised*…* (P4, Consultant in diabetes)

### Impact of the digital divide in healthcare practice

HCP experiences during COVID-19 provided a useful example to reflect on the potential impact of digital services on health inequalities:

… at one point our weight loss and diabetes prevention services were purely digital and I only had that to offer and that really made me worry that I'm only giving these people one option and it might not be the appropriate one. (P8, GP)

Participants shared their observations of significant factors relating to digital health inequalities in practice; less focused on age and ethnicity; and more related to individuals’ digital skills, language skills, and trust and familiarity with DHIs and the healthcare system. Additionally, cost and lack of privacy through the use of a shared device impacted DHI uptake. HCPs spoke about how existing pressures, such as short appointment times, made it difficult to assess or provide healthcare users with appropriate information about digital services.

Because I think we're kind of expected to assess people’s digital literacy or their preference before we refer them or suggest. But often you don't either have time, or you can't - you just kind of have to get on and make the suggestion or make the referral. (P8, GP)

Participants felt that both digital and non-digital interventions had the potential to exacerbate existing inequalities. For example, the lack of tailoring of advice and guidance to different cultural groups and the lack of information about appropriate community resources such as social prescribing excluded some groups from benefiting. From the opposite perspective, it was felt that those who benefitted the most from digital were those who already took positive action in relation to their health.

The people I would see in clinic would be the ones who would probably like to engage a bit more and so you would get them (using DHIs). The ones who wouldn't turn up… perhaps you would miss a lot of them and that would be a lot more minorities. (P10, Consultant in diabetes)

While it was felt that service commissioners were recognising the higher risk of health inequalities experienced by some ethnic minority individuals, the lack of data around DHI implementation, uptake and use made it difficult to understand the full picture of inequalities.

I do wonder if there’s something around intersectionality… there are other challenges that people face within health, within healthcare… it’s very rare that somebody… has only one challenge… So if you overlay disability, and you know, and learning difficulties and other things into the mix, and I don't know that there’s been enough data captured - there may be subsets of people that are disadvantaged or missed, it may just have been that they've not been offered… I don't know if that data is captured so that anybody knows – because you don't know what you don't know, do you? (P3, Pharmacist)

Overall, participants showed both concern and optimism about the potential of DHIs in relation to health inequalities. A key concern was that over time, services would rely more routinely on digital services, reducing the quality of care and excluding some demographic groups. Others suggested that digital approaches could improve access to healthcare for some people who would struggle to attend, such as those who found it difficult to leave the house, as well as helping overcome language barriers through videos rather than written resources. Participants also shared some examples of good practice to engage their local population in DHIs and programmes around diabetes management.

We've been running (structured diabetes education) online via teams, and… I had long conversations with our education team saying you know, are we really going to run this this way and is this the only way we're going to offer education now? And you know, what about the people who are going to miss out? And again the engagement with education has been really, really positive amongst our South Asians… amongst groups that I wouldn't have assumed would be very (IT familiar)… What I'm slightly worried about is that it will end up being the only offer that we have. (P4, Consultant in diabetes)

… Desmond, which is a diabetes education support program. We've had a local voluntary service provide that in Urdu and Punjabi as well. Previously, we couldn't refer to the structured diabetes education program because it was only delivered in English. So for a lot of our patients, that was no good. That’s changed. (P6, GP)

### HCP recommendations for equitable uptake of DHIs

Participants made recommendations related to the equitable uptake of DHIs in three areas: design, implementation and engaging populations experiencing or at risk of health inequalities through the process. Recommendations around design focused on improving accessibility of DHIs for individuals with a range of access needs, such as using diagrams, simple language and audio and video options, and improving cultural appropriateness of content (eg, around food information in healthy living interventions). They highlighted that some systems charge to add additional languages, and while this might be too expensive at an NHS Trust level, it could be affordable at a national level. They also drew attention to the need to improve communication of legal, data protection and permissions information in a way that was understandable to the public. To maintain usage, the DHI offer should be tailored to individual interest, and the intervention should not be too complex or time-consuming.

It helps to be culturally specific… And by that what we mean is - talking in that cultural language. So perhaps using certain words or using certain examples of foods, not just translating things, but it’s much deeper than that. (P16, Dietician)

Participants recommended the use of low-tech solutions, such as SMS messaging, as a ‘universal approach’, although it was emphasised that DHIs should only be part of a range of options and non-digital services were essential to reach everyone. To support the implementation of DHIs more generally, clinicians wanted to see more evidence-based recommendations from trusted organisations such as national charities and commissioning from the NHS, and education and support for HCPs, including those in pharmacy and new primary and social care roles, to enable them to support healthcare users to select and use DHIs for their health.

Participants felt that there were opportunities to bridge the digital divide. They recommended engaging with healthcare users and the community to understand digital access and literacy needs and working with existing community structures. This included drawing on family support to introduce virtual consultations or DHIs, particularly where younger generations had healthcare training; using community interest in health as an information network; providing information via cultural media; and working with local champions. They also highlighted the need to be sensitive to the expectations of the local population in relation to the role of different parts of the clinical team and providing education to improve uptake of appointments with the complement of healthcare professions. To support continued use and benefit from DHIs, participants highlighted the need to think holistically and engage the community to address the facilitators and barriers of the behavioural change approach, rather than just the DHI. They emphasised the need for follow-up and for joint working between primary care, pharmacy, local government and voluntary organisations to deliver equitable care.

I think health care professionals or community leaders recommending them and supporting them to use it can help with ongoing use as well. So having that kind of check in with people to ask, are you still using it? How are you finding it? (P8, GP)

A big part (of supporting people to engage or download their first app) would be played by sort of voluntary and tertiary sector organizations… who are trusted by the community … I think some of it would be (HCPs) in the community… so an element of their role would be to increase engagement with IT solutions, to support self-care and management of chronic conditions. (P6, GP)

## Discussion

### Principal findings

HCPs appreciated the potential benefits of DHIs to improve access and delivery of care and healthcare user outcomes. Barriers to implementing DHIs in practice included a need for a repository of trusted DHIs, a lack of time to introduce and support DHIs to healthcare users and a need for additional training and support. Secondly, HCPs had a good understanding of the challenges individuals faced in relation to wider inequalities, barriers to healthy behaviours and healthcare access and digital health. They were concerned that over-reliance on digital interventions within the healthcare system may exacerbate existing inequalities. HCPs identified that groups that are particularly at risk of digital exclusion include those experiencing deprivation, individuals who did not speak English and/or with low literacy, people with learning difficulties and those with physical impairments that might impact the use of particular tools, for example, sight, hearing and arthritis. Third, participants made recommendations about how the health system can improve the digital offer, through design, implementation approach and engaging populations experiencing or at risk of health inequalities. Finally, participants emphasised the importance of ensuring non-digital services remained available to ensure equitable access to care.

### Comparison with prior work

Previous studies looking at HCP views on acceptability of the implementation of digital in health have also reported ambivalence and described both opportunities, for example, increased healthcare user self-management and concerns, such as around usability, privacy and cost.^10^ Common barriers to adoption by HCPs include individual factors (such as confidence in prescribing digital interventions), impact on practice (eg, time and resource implications) and intervention factors (including lack of evidence for effectiveness and security concerns).^9 22^

Clinicians also reported infrastructural barriers, suggesting that centralised commissioning would provide assurance and address additional costs associated with improving accessibility of DHIs. Centralised systems for DHI reimbursement are in place to some extent across Europe,^23^ but evaluation of the DiGA ‘app on prescription system’ in Germany suggests that there is an emerging divide in DHI uptake.^24^ HCP reflections on the success of digital interventions (such as digital weight loss programmes) were in line with evaluations of these programmes.^25 26^

Clinicians reflected both on the enabling impact of the pandemic on both public and healthcare system openness towards digital health and its potential impact in worsening inequalities in care access and outcomes. For example, some healthcare users were able to use commercially available apps to support diagnosis of atrial fibrillation. Other research at this time described COVID-19 as a destabilising experience for healthcare providers and noted that there had been a lack of cultural change to deal with the introduction of telehealth.^27^ Additionally, HCPs in our study discussed how their previous perceptions of who might use DHIs were challenged by the uptake during this time, particularly in relation to older adults. Previous work has highlighted that when HCPs hold stigmatising attitudes about ageing, this can influence the use and adoption of DHIs,^28^ and that the gap between willingness to use and recommendations from HCPs increases with age^29^; this suggests that providing education and support to HCPs to recommend DHIs to a wider range of people may increase uptake in those that might benefit.

In addition to reducing inequalities in how DHIs are offered, participants also suggested that digital approaches might enable more accessible care, for example, through the use of video rather than written sources, reducing the need for travel for those who had financial and health barriers to in-person access and being more practical for those who might not be able to get time off work. Other studies have also highlighted how DHIs might improve the provision of culturally sensitive information, for example, HCPs providing food advice to women from diverse backgrounds with gestational diabetes found a culturally sensitive DHI could fill gaps in their knowledge about other food cultures^30^, and others have suggested that advances in artificial intelligence could improve health information access for linguistically diverse populations, including through real-time translation.^31^

Findings from these interviews with HCPs reflect our discussions with healthcare users, who described the negative impact of digitisation on healthcare access, and experiences of digital barriers due to individual characteristics, awareness and support. Healthcare users highlighted the role of HCPs and via community organisations as trusted sources to enable individuals to access and use DHIs.^32 33^ Findings from across these studies were discussed with stakeholders to develop a list of prioritised recommendations for actions at the individual, intervention, healthcare provider and policy level.^34^

### Limitations

This study used both focus groups and interviews: this may have influenced what participants felt able to share (in a group setting) or prompted less reflection in relation to others’ practice (in individual interviews). However, this pragmatic approach ensured we could include a range of HCPs, and similar statements emerged from both data collection methods. Inclusion of HCPs from a range of settings provides a useful overview of the challenges and opportunities across the health system (eg, regarding training and commissioning) and at different parts of the healthcare user journey (such as prevention and specialist management). Future work should focus on how suggested approaches to equitable implementation of digital health will impact specific professions, such as community pharmacists.

The majority of participants in the study were from a South Asian ethnic background (n=13, 72%). This may partly reflect the composition of the clinical workforce (42% from black and minority ethnic backgrounds in 2020)^35^ and interest or confidence in speaking about ethnic inequalities in digital health. One focus group participant was concerned about using incorrect phrasing and unintentionally causing offence. A Royal College of Physicians’ report on addressing health inequalities in practice found that 67% of clinicians feel they had not received enough training, and only 31% felt confident in their ability to talk to healthcare users about the impact of inequalities on their health.^36^ Additionally, most participants were under 50 years of age, and previous studies have found that HCPs aged over 50 were less likely to use digital devices and more likely to report lower digital confidence.^37^ A Health Education England review found that many NHS staff reported no training for digital transformation.^38^ This may have impacted participant confidence in speaking about digital inequalities experienced by healthcare users. We addressed this through creating a supportive space for discussion and offering participants individual interviews if they preferred.

Participants’ experience with digital cardiometabolic interventions also varied by role; for example, those working in secondary care had more experience with tools such as remote monitoring of atrial fibrillation or blood glucose, while those in primary care spoke about tools to do with lifestyle change, information provision and access to services.

While this study focused on South Asians, as the largest minority ethnic group in the UK,^39^ the findings highlighted that approaching the question of DHI solutions via ‘ethnicity’ or other broad social groups was not always considered suitable for real-life practice. Participants spoke instead about barriers to access experienced by individuals (such as literacy, financial, learning disabilities and physical impairments that could impair smartphone use) or about social barriers to engagement with healthcare, such as language and culture in specific communities in their area. This is reflected in the recommendations on improving DHI design and implementation to improve accessibility and utility for all.

## Conclusion

This study describes HCP perspectives on digital health inequalities with a focus on cardiometabolic diseases. HCPs described the complexities of delivering care to underserved communities and the potential for digital approaches to both address and exacerbate inequalities. Participants provided recommendations related to design, implementation and engaging target populations, providing practical examples to address digital health inequalities. Particular emphasis was given to the need for better NHS evaluation and commissioning to support HCPs to use DHIs in practice.

## supplementary material

10.1136/bmjopen-2024-091018 online supplemental file 1

## Data Availability

No data are available.
