# Supplementary material for: Healthcare professionals’ attitudes towards digital health interventions and perspectives on digital health inequalities in cardiometabolic care: a qualitative study
Source: BMJ Open. 2025 Feb 26;15(2):e091018. doi: 10.1136/bmjopen-2024-091018 (PMC11865753; doi:10.1136/bmjopen-2024-091018)
Supplement: online supplemental file 1 [file bmjopen-15-2-s001.docx]

# Supplementary 1: Focus group guide for HCPs: barriers and facilitators to uptake of DHIs at an individual and intervention level

## NB: Schedules were adapted for individual interviews and according to the professional composition of the group

| Study and participant introduction | - Welcome - Permission to record - Participant introductions - Introduction of focus group aims and outline of the session - Online session ‘rules’ |
| --- | --- |
| DHIs introduction | - Definition of DHIs - Allow time for questions before start |
| *Part 1* | *In this section we seek to understand use of DHIs in South Asian populations, and for CVD and DM.* |
| Professional experience of DHIs for CV or DM | - What professional experience do you have with DHIs for cardiovascular disease or diabetes (or for other health problems)?   *Prompts:*   - - *E.g. working at front-line with patients on implementation and use, developing technology, or other?*   - *If no, what is your professional experience related to DHIs or CMD?* - In your experience, how and how often are DHIs used for CV/DM? - Do these DHIs replace, enhance or provide additional care and support for patients? |
| Barriers and facilitators to use | In this section we will focus on barriers and facilitators to use. You can answer in reference to: DHIs you have experience of or other DHIs which have been used in cardiovascular disease, diabetes, or which affect these patient groups.   - In your opinion, what features of DHIs for CV or DM affect their accessibility and usability to patients and the public?   - *What are the facilitators or* barriers?   - What features affect how a DHI delivers its intended outcomes? - As a health professional, what factors impact whether you recommend use of a particular DHI to a patient?   - *For other HCPs, what is your experience/interactions with patients related to DHIs for CMD/CMD management?*   - *Which apps have been more or less useful in the long-term?* - In your experience, what other reasons are there for end users to want or not want to use DHIs? |
| Population differences in use of DHIs | Wider population   - Are you aware of any patterns of differences in use of DHIs, for example by age, socioeconomic status, or ethnicity?   - *What do you think are the reasons for these differences?*   - *As use of DHIs becomes more widespread in health and care, which groups do you think will have greater or less access?*   Population – South Asian   - Focussing on experience of DHIs of those of a South Asian background, can you share your experience or opinion on whether particular DHIs have been successful or unsuccessful in South Asian communities?   - *What about these made them successful/unsuccessful?*   Population - inequalities   - What types of alternatives can you offer those without digital access? - Can you tell me about anything that could help people in these groups use DHIs (that we haven’t already discussed)? |
| *Part 2* | *In this section we will explore the impact of the coronavirus pandemic on use of DHIs. Recognise that this is a sensitive topic for many individuals, and if people feel the need to withdraw at any point in the discussion they can do so.* |
| Coronavirus impact on health management | - How has the coronavirus pandemic changed the way you support patients?   - *to manage CVD/DM?*   - *changes in use to DHIs?* |
| Use of coronavirus DHIs/  Coronavirus and inequalities | - In your experience, how have patients and the public used technology to get information about coronavirus, or to take any action related to coronavirus (like testing or vaccination)? - *If applicable to your area of work, were you able to offer official information about coronavirus or about action, such as testing or vaccination, in ways that are not digital?* - *If specific information related to coronavirus and CVD/DM was available, were the formats appropriate to your patient population?* - In your experience or opinion, have there been inequalities arising from the use of technology in healthcare during coronavirus?   - *Were there any specific patterns of use that you found interesting or concerning? (Any specific to South Asian populations?)* |
| Wrap-up | - Summarise key points in each topic - Any new or closing comments from participants |
| Session close | - Thank for participation - Signpost to consent form regarding consent, data protection, outputs of session and contacting research team - Remind participants that they will receive their £50 voucher and a sources of further support sheet in the post. |
