# Supplementary material for: Healthcare professionals’ attitudes towards digital health interventions and perspectives on digital health inequalities in cardiometabolic care: a qualitative study
Source: BMJ Open. 2025 Feb 26;15(2):e091018. doi: 10.1136/bmjopen-2024-091018 (PMC11865753; doi:10.1136/bmjopen-2024-091018)
Supplement: Supplementary file 2 [file bmjopen-2024-091018.reviewer_comments.pdf]

PEER REVIEW HISTORY

BMJ Open publishes all reviews undertaken for accepted manuscripts. Reviewers are asked to complete a checklist review form and are provided with free text boxes to elaborate on their assessment. These free text comments are reproduced below.

ARTICLE DETAILS

Title (Provisional)

Healthcare professionals' attitudes towards digital health interventions and perspectives on digital health inequalities in cardiometabolic care: a qualitative study

Authors

Ramasawmy, Mel; Sunkersing, David; Poole, Lydia; Blandford, Ann; Gill, Paramjit; Khunti, Kamlesh; Modha, Shivali; Patel, Kiran; Potts, Henry; Sajid, Madiha; Khan, Nushrat; Banerjee, Amitava

---

VERSION 1 - REVIEW

**Reviewer**	**1**
**Name**	**Halms, Theresa**
**Affiliation**	**University of Augsburg**
**Date**	**06-Sep-2024**
**COI**	**None**

Thank you for the opportunity to review this paper. The study provides valuable insights into potential barriers and facilitators to the implementation of digital health interventions and, due to its qualitative approach, highlights aspects that were not considered previously.

• Introduction:

o The authors state that the South Asian population experiences poorer cardiometabolic health outcomes and are potentially more likely to face barriers to digital inclusion. Given that this study aims to investigate healthcare professionals' views on the implementation of digital health interventions and not the patients' perspective, it remains unclear to me why the HCPs' ethnicity is of relevance.

o I believe it might enhance the comprehensibility of the paper if the authors shared their definition of digital health interventions

• Methods:

o The description of the study's objective suggests that South Asian participants were deliberately recruited; however, in the Methods section there is no mention of a targeted

recruitment of this ethnic group. If the rather large proportion of South Asian interviewees was unintentional, I believe it is important for the authors to make this clear.

o It might be helpful if the authors specified the data collection method (e.g. semi-structured interviews). Did the authors utilize member checking to enhance the credibility of the results?

• Results:

o Could the authors please provide information on the number of conducted focus groups and the respective number of participants as well as on the number of individual interviews?

o Were the authors able to identify differences between the aspects discussed during the focus group interviews and the individual interviews? If so, it would be interesting to elaborate on these.

• Discussion:

o Given the association between age and affinity with digital tools, the fact that only two participants were over the age of 50 should be mentioned as a limitation

o Further, the different methodological approaches (focus groups and individual interviews) represent an important limitation. Group interviews may have increased the likelihood of social desirability bias and thus made controversial statements less likely, while individual interviews offered fewer oppportunities for discussion.

o The study's results and participants' statements frequently address their impressions of patients and their experiences, barriers, etc. with DHI. I therefore think that the potential discrepancy between the perceptions of the participating HCPs and those affected requires discussion.

**Reviewer**	**2**
**Name**	**Aryal, K.**
**Affiliation**	**McMaster University, HEI**
**Date**	**24-Sep-2024**
**COI**	**NA**

Abstract:

1. Very long and confusing sentence: "Online interviews and focus-groups with HCPs (n=18), exploring participants' experiences and attitudes towards digital health, perceptions of patients' barriers and facilitators to use, whether they perceived any populations to be particularly at risk of digital inequalities, and the impact of the COVID-19 pandemic on their practice in relation to digital health."

2. Unclear statement “Participant recommendations related to design, implementation, and engaging target populations providing practical examples to address digital health inequalities, such as working with other sectors to take a community approach.”

Introduction:

1. In the first paragraph you should clearly define what DHIs are. I think you have tried to do it on line 33-34 but it should be more explicit.
2. This sentence is too long – you need to break it up “Previous studies have shown variation in the digital health competence of HCPs [7], and highlighted barriers and facilitators to HCP use of digital health, including infrastructure, technical barriers, training, evidence about technology effectiveness, concerns about workload, and individual personal and psychological barriers, such as resistance to change or concerns about losing human interaction [8]”
3. Overall good introduction – there are some grammatical errors that need to be fixed.

Methods

1. Another run-on sentence: “The discussions explored professionals’ experiences and attitudes towards digital health, perceptions of patients’ barriers and facilitators to use, whether they perceived any populations to be particularly at risk of digital inequalities, and the impact of the COVID-19 pandemic on their practice in relation to digital health (full details are provided in Supplementary material 1).”

Results:

1. Have some subheadings in the results and improve the organization of the results
2. Could you have done a comparison between South Asian and non-South Asian responses?

Discussion:

1. Maybe add or consider discussing next steps such as quantitative analysis on DHI use or qualitative analysis from a patient perspective on DHI use

Overall, really interesting work!

**Reviewer**	**3**
**Name**	**Laur, Celia**
**Affiliation**	**Women’s College Hospital**
**Date**	**07-Oct-2024**
**COI**	**None to declare.**

Healthcare professionals' attitudes towards digital health interventions and perspectives on digital health inequalities: a qualitative study

This paper aims to explore the perceptions of healthcare professionals on the use of digital health innovations among the South Asian population in the UK, specifically for cardiometabolic diseases. Although this focus seems quite specific, the participants are from a broad range of professionals (GPs, pharmacists, dietitians etc.). There is some detail missing from the methods, as explained below. Overall, further highlighting why this paper is needed, and what it adds to the field will help distinguish this work from the many other studies that have already been done and found similar results.

Introduction

- Please define what you mean by Digital Health Interventions, and the breadth of interventions that this could include. For example, would an EMR or a patient portal be included in your definition, or are you more focused on apps and mobile devises like smartwatches?

- Your introduction can make a stronger case for why this study was needed. Why are you looking at multiple healthcare professions together rather than focused on one? Why specifically cardiometabolic conditions for DHIs? Why are you focused specifically on the South Asian population? There has been a lot of work done on DHIs, so you'll need to make a strong case for why this work is unique. The one sentence justification included currently is not sufficient to make this case.

Methods

Details are missing from the methods. Please double check you COREQ checklist and make sure all information is included. I've included a few suggestions for additional detail to include.

- Please provide more detail regarding your recruitment strategy so it could be more likely to be reproducible.

- Details on if the interviewer knew the participants, and characteristics of the interviewer are said to be in the Supplemental files, but I did not see those details. A positionality statement from the authors would be important to include in the main text, including their relationship and understanding of South Asian culture, as this may impact the interpretation of results.

- Further detail on the analysis approach taken, including the coding process, is needed. How was the coding completed? Even if it was done in Word, that still needs to be included. Mention if it was pilot tested in the methods (COREQ says supplement, but again I couldn't find that information).

- Data saturation should be discussed in the methods. COREQ says it is n/a, but given your approach, there should be some mention of when interviews were stopped.

- Further detail on the coding process would strengthen the methods section.

- Please specify if participants were able to comment on a summary of results before publication. Or if any other methods of member checking were completed.

Results

- To include the additional details requested for the methods and discussion, yet keep within the word count, you might consider moving some of your quotes into a Table rather than having all of them in the text.

- On page 4/5, I don't understand the quote which includes "chance them away"? Please add more clarity or select a different quote to represent your point as I'm not sure what this one means.

- On page 5 you include references for other research. Please move those comments and references to the discussion so the results are only reflective of your work. You mention that there is work with patients, so you may want to move that to the discussion and connect it with a broader point about how this work needs to be complemented with the patient perspective.

- Within your results, please make sure all results relate back to your research question. There are some sections, including the top of "Perceptions of inequalities..." which seem more generic and, to me, do not necessarily relate to your specific question. Clarification of the connection is recommended (not changing the results).

Discussion

- In the limitations, please acknowledge the challenges of including such a wide range of professionals, including in terms of planning for next steps. For example, with this broad approach it is difficult to know if similar strategies are needed for pharmacists as for dietitians or GPs. You've also included primary and secondary care together which makes it even more broad and difficult to apply the results.

- In the discussion, you'll need to acknowledge that you are only focused on HCP perspectives, yet this question really needs to be considered in connection with patient perspectives. I believe you mention work with patients is underway and it would be beneficial to explain if these HCP results should/can be considered alongside patient interviews which were also done/underway/needed.

- Please acknowledge the implications of having 72% of your participants identify as South Asian. Was this planned (if so, this needs to be included in the methods)? With these participants, it means you are mainly hearing the perspectives of those who are South Asian and may have a more personal connection to the culture, etc. as described. For those that are not South Asian, the experience could be quite different. How did you account for this difference in your analysis (if you didn't, why not)? What might this mean for your results and how they are used?

- Please add more about the impact of these results. How can they be applied? How does knowing this information support more equitable approaches to DHI use? What questions remain?

VERSION 1 - AUTHOR RESPONSE

Reviewer: 1
Dr. Theresa Halms, University of Augsburg
Comments to the Author:
Thank you for the opportunity to review this paper. The study provides valuable insights into potential barriers and facilitators to the implementation of digital health interventions and, due to its qualitative approach, highlights aspects that were not considered previously.

We thank the reviewer for their time and suggestions for improvement.

• Introduction:
o The authors state that the South Asian population experiences poorer cardiometabolic health outcomes and are potentially more likely to face barriers to digital inclusion. Given that this study aims to investigate healthcare professionals' views on the implementation of digital health interventions and not the patients' perspective, it remains unclear to me why the HCPs' ethnicity is of relevance.

➢ Thank you for this comment. These interviews were conducted in the context of a study about digital health inequalities faced by South Asian patients in the UK. We have clarified this paragraph to demonstrate that this line refers to South Asian patients rather than HCPs (p2). Elsewhere we discuss the possible significance of HCP ethnicity in relation to their contributions to the discussion (p12).

o I believe it might enhance the comprehensibility of the paper if the authors shared their definition of digital health interventions

➢ Thank you. We have included our definition in the background (p2).

• Methods:
o The description of the study's objective suggests that South Asian participants were deliberately recruited; however, in the Methods section there is no mention of a targeted recruitment of this ethnic group. If the rather large proportion of South Asian interviewees was unintentional, I believe it is important for the authors to make this clear.

- ➢ Thank you. We did not deliberately recruit South Asian participants, but have commented on the potential reasons for this in the description of participant characteristics (p3) and the impact on our findings in the limitations (p12).

o It might be helpful if the authors specified the data collection method (e.g. semi-structured interviews). Did the authors utilize member checking to enhance the credibility of the results?

- ➢ In the methods section we highlight that we invited participants to focus groups first, but if they were unavailable we offered interviews (p3). This was to ensure that HCPs could participate at a time that was convenient to them. We have included a line to clarify that these were semi-structured, and that key ideas were summarised and confirmed by participants during the interview (p3).

• Results:

o Could the authors please provide information on the number of conducted focus groups and the respective number of participants as well as on the number of individual interviews?

- ➢ This has now been included in the 'participant characteristics' section (p3).

o Were the authors able to identify differences between the aspects discussed during the focus group interviews and the individual interviews? If so, it would be interesting to elaborate on these.

- ➢ Thank you for this comment. In general, the individual interviews focused more on their specific areas of experience, while the focus groups allowed for more questions between participants, but there were no significant differences in the aspects discussed.

• Discussion:

o Given the association between age and affinity with digital tools, the fact that only two participants were over the age of 50 should be mentioned as a limitation

- ➢ Thank you for raising this issue. We have included this in our limitations (p12).

o Further, the different methodological approaches (focus groups and individual interviews) represent an important limitation. Group interviews may have increased the likelihood of social desirability bias and thus made controversial statements less likely, while individual interviews offered fewer oppportunities for discussion.

- ➢ We have included some reflection on the pragmatic approach taken to data collection in the limitation section (p12).

o The study's results and participants' statements frequently address their impressions of patients and their experiences, barriers, etc. with DHI. I therefore think that the potential discrepancy between the perceptions of the participating HCPs and those affected requires discussion.

- ➢ Comparisons to our related work with individuals with cardiometabolic disease from a South Asian background have been included in discussion (p11-12).

Reviewer: 2
Dr. K. Aryal, McMaster University
Comments to the Author:
Well written.
Please see comments attached. (BMJOpen Review.pdf)

We thank the reviewer for their time and suggestions for improvement.

Abstract:

1. Very long and confusing sentence: "Online interviews and focus-groups with HCPs (n=18), exploring participants' experiences and attitudes towards digital health, perceptions of patients' barriers and facilitators to use, whether they perceived any populations to be particularly at risk of digital inequalities, and the impact of the COVID-19 pandemic on their practice in relation to digital health."

- Thank you for this feedback. We have revised the abstract in line with comments from the editor and reduced the length of this sentence.

2. Unclear statement "Participant recommendations related to design, implementation, and engaging target populations providing practical examples to address digital health inequalities, such as working with other sectors to take a community approach."

- We have rephrased this line to clarify the recommendations made by participants.

Introduction:

1. In the first paragraph you should clearly define what DHIs are. I think you have tried to do it on line 33-34 but it should be more explicit.

- Thank you, we have made this more explicit at the start of the background (p2).

2. This sentence is too long – you need to break it up "Previous studies have shown variation in the digital health competence of HCPs [7], and highlighted barriers and facilitators to HCP use of digital health, including infrastructure, technical barriers, training, evidence about technology effectiveness, concerns about workload, and individual personal and psychological barriers, such as resistance to change or concerns about losing human interaction [8]"

3. Overall good introduction – there are some grammatical errors that need to be fixed.

Methods

1. Another run-on sentence: "The discussions explored professionals' experiences and attitudes towards digital health, perceptions of patients' barriers and facilitators to use, whether they perceived any populations to be particularly at risk of digital inequalities, and the impact of the COVID-19 pandemic on their practice in relation to digital health (full details are provided in Supplementary material 1)."

- Thank you. We have split these sentences (p2 and 3) and reviewed the paper for grammatical errors.

Results:

1. Have some subheadings in the results and improve the organization of the results

- Thank you. We have added some sub-headings in the results and edited these sections to focus on the findings that relate to the research question around health inequalities (p5 onwards).

2. Could you have done a comparison between South Asian and non-South Asian responses?

- ➢ Thank you for this suggestion. We discussed this approach during analysis, but found that HCPs emphasised the nature of inequalities they observed as being more related to social deprivation than specific ethnic or cultural groups. Where it was relevant we included some details about HCP ethnicity and how this related to their practice (e.g. p7-8) as well as how it may have impacted individuals' confidence in broaching these issues (p12).

Discussion:

1. Maybe add or consider discussing next steps such as quantitative analysis on DHI use or qualitative analysis from a patient perspective on DHI use

- ➢ We have included some discussion around patient perspectives based on other work conducted as part of this study in the 'comparison with prior work' section (p11-12).

Overall, really interesting work!

Reviewer: 3
Dr. Celia Laur, Women's College Hospital , University of Toronto
Comments to the Author:
Please see attached. (BMJ Open Review-Oct6,2024.pdf)

This paper aims to explore the perceptions of healthcare professionals on the use of digital health innovations among the South Asian population in the UK, specifically for cardiometabolic diseases. Although this focus seems quite specific, the participants are from a broad range of professionals (GPs, pharmacists, dietitians etc.). There is some detail missing from the methods, as explained below. Overall, further highlighting why this paper is needed, and what it adds to the field will help distinguish this work from the many other studies that have already been done and found similar results.

- ➢ We thank the reviewer for their suggestions on how we can strengthen the paper.

Introduction

• Please define what you mean by Digital Health Interventions, and the breadth of interventions that this could include. For example, would an EMR or a patient portal be included in your definition, or are you more focused on apps and mobile devises like smartwatches?

- ➢ Thank you, we have made this more explicit at the start of the background (p2).

• Your introduction can make a stronger case for why this study was needed. Why are you looking at multiple healthcare professions together rather than focused on one? Why specifically cardiometabolic conditions for DHIs? Why are you focused specifically on the South Asian population? There has been a lot of work done on DHIs, so you'll need to make a strong case for why this work is unique. The one sentence justification included currently is not sufficient to make this case.

- ➢ Thank you, this forms part of a larger study looking at digital health inequalities in cardiometabolic disease, using the South Asian population in the UK as a case study. We have made our reasoning more explicit in this paragraph (p2).

Methods

Details are missing from the methods. Please double check you COREQ checklist and make sure all information is included. I've included a few suggestions for additional detail to include.

• Please provide more detail regarding your recruitment strategy so it could be more likely to be reproducible.

- We have provided additional detail about the routes taken to circulate the study advert (p3).

• Details on if the interviewer knew the participants, and characteristics of the interviewer are said to be in the Supplemental files, but I did not see those details. A positionality statement from the authors would be important to include in the main text, including their relationship and understanding of South Asian culture, as this may impact the interpretation of results.

- We apologise for the oversights related to submission of the supplementary material related to COREQ. This information has now been included in the methods section instead.
- We have included a positionality statement in the methods regarding authors MR, NK and DS, and about the wider co-author group (p3).

• Further detail on the analysis approach taken, including the coding process, is needed. How was the coding completed? Even if it was done in Word, that still needs to be included. Mention if it was pilot tested in the methods (COREQ says supplement, but again I couldn't find that information).

• Further detail on the coding process would strengthen the methods section.

- We have included some additional information on how the coding was completed (p3).

• Data saturation should be discussed in the methods. COREQ says it is n/a, but given your approach, there should be some mention of when interviews were stopped.

- We have included details on when interviews were stopped (p3).

• Please specify if participants were able to comment on a summary of results before publication. Or if any other methods of member checking were completed.

- Participants were not invited to comment on this paper, however health care professionals were invited to attend a stakeholder event where study findings were presented for discussion. This is due for publication shortly.
- Member checking was conducted during the discussions, details on this have been included (p3).

Results

• To include the additional details requested for the methods and discussion, yet keep within the word count, you might consider moving some of your quotes into a Table rather than having all of them in the text.

- Thank you for this suggestion: we have made some edits to the quotes to maintain the word count. Previous reviews have suggested reader preference for quotes to be in-text.

• On page 4/5, I don't understand the quote which includes "chance them away"? Please add more clarity or select a different quote to represent your point as I'm not sure what this one means.

- ➢ We’re not sure which quote this is referring to, but we have reviewed the quotes and added clarify where required.

• On page 5 you include references for other research. Please move those comments and references to the discussion so the results are only reflective of your work. You mention that there is work with patients, so you may want to move that to the discussion and connect it with a broader point about how this work needs to be complemented with the patient perspective.

- ➢ Thank you for this comment. We have kept some references in text (e.g. where they explain particular programmes mentioned by participants) and moved others to the discussion section (p11).

• Within your results, please make sure all results relate back to your research question. There are some sections, including the top of “Perceptions of inequalities…” which seem more generic and, to me, do not necessarily relate to your specific question. Clarification of the connection is recommended (not changing the results).

- ➢ We note this recommendation and have reviewed the results section, directing its relevance to the research question where need.

Discussion

• In the limitations, please acknowledge the challenges of including such a wide range of professionals, including in terms of planning for next steps. For example, with this broad approach it is difficult to know if similar strategies are needed for pharmacists as for dietitians or GPs. You’ve also included primary and secondary care together which makes it even more broad and difficult to apply the results.

- ➢ Our approach was to capture a broad range of HCP understanding of digital inequalities. We have added a discussion of this to the limitation section (p12).

• In the discussion, you’ll need to acknowledge that you are only focused on HCP perspectives, yet this question really needs to be considered in connection with patient perspectives. I believe you mention work with patients is underway and it would be beneficial to explain if these HCP results should/can be considered alongside patient interviews which were also done/underway/needed.

- ➢ Thank you for this suggestion. We have included some additional information about our studies of patient perspectives and next steps (p12).

• Please acknowledge the implications of having 72% of your participants identify as South Asian. Was this planned (if so, this needs to be included in the methods)? With these participants, it means you are mainly hearing the perspectives of those who are South Asian and may have a more personal connection to the culture, etc. as described. For those that are not South Asian, the experience could be quite different. How did you account for this difference in your analysis (if you didn’t, why not)? What might this mean for your results and how they are used?

- ➢ The high level of recruitment of participants from a South Asian background was not planned (see ‘participant characteristics’ on p4) for an explanation.
- ➢ Thank you for this suggestion, which was also raised by reviewer 2. We found that across the discussions, HCPs emphasised the nature of inequalities they observed as being more related to social deprivation than specific ethnic or cultural groups. Where it was relevant we have included some details about HCP ethnicity and how this related to

their practice (e.g. p7-8). We have also discussed how it may have impacted individuals' confidence in broaching these issues (p12).

• Please add more about the impact of these results. How can they be applied? How does knowing this information support more equitable approaches to DHI use? What questions remain?

- We have included some additional information about our studies of patient perspectives and next steps (p12).

VERSION 2 - REVIEW

**Reviewer**	**1**
**Name**	**Halms, Theresa**
**Affiliation**	**University of Augsburg**
**Date**	**22-Jan-2025**
**COI**	

I have no further suggestions for improvement and would recommend the paper for acceptance in its current form.

Thank you for the opportunity to review this manuscript and congratulations to the authors on their important work.

**Reviewer**	**3**
**Name**	**Laur, Celia**
**Affiliation**	**Women's College Hospital**
**Date**	**21-Jan-2025**
**COI**	

Great work on addressing the changes!
